# Associations of lower values of peak oxygen uptake and handgrip strength with a smaller liver volume

**DOI:** 10.48101/ujms.v130.11924

**Published:** 2025-05-16

**Authors:** Muhammad Naeem, Marcello Ricardo Paulista Markus, Martin Bahls, Mohammed Mousa, Marcus Dörr, Jens-Peter Kühn, Robin Bülow, Stephan B. Felix, Giovanni Targher, Beate Stubbe, Ralf Ewert, Henry Völzke, Till Ittermann

**Affiliations:** a Institute for Community Medicine, SHIP/Department of Clinical Epidemiology, Greifswald, Germany; b Department of Zoology, University of Malakand, Chakdara Dir (L), Pakistan; c Department of Internal Medicine B – Cardiology, Intensive Care, Pulmonary Medicine and Infectious Diseases, Greifswald, Germany; d German Center for Diabetes Research (DZD), Partner Site Greifswald, Greifswald, Germany; e DZHK (German Center for Cardiovascular Research), Partner Site Greifswald, Germany; f Institute and Policlinic for Diagnostic and Interventional Neuroradiology, University Hospital, Carl Gustav Carus University, TU Dresden, Dresden, Germany; g Institute of Diagnostic Radiology and Neuroradiology, University Medicine Greifswald, Greifswald, Germany; h Department of Medicine, University of Verona, Verona, Italy; i Metabolic Diseases Research Unit, IRCCS Sacro Cuore - Don Calabria Hospital, Negrar di Valpolicella, Italy; j DZD (German Center for Diabetes Research), Site Greifswald; Germany

**Keywords:** Peak oxygen uptake, handgrip strength, liver volume

## Abstract

**Background and aims:**

The associations between physical fitness markers and liver volume in the general population are unclear. We investigated the associations of peak oxygen uptake (VO_2peak_)and handgrip strength with liver volume in a general population sample.

**Methods and results:**

Data were taken from 1,531 German adults (51.3% women), aged 20 to 88 years, from two cohorts of the population-based Study of Health in Pomerania (SHIP-START-2 and SHIP-TREND-0). We analysed cross-sectional associations of VO_2peak_ and handgrip strength with liver volume derived from magnetic resonance imaging (MRI) by using multivariable linear regression models. These models were adjusted for age, sex, body fat mass, pre-existing type 2 diabetes, daily alcohol consumption, smoking status, and use of hypoglycaemic or antihypertensive medications. We observed significant associations of lower VO_2peak_ and handgrip strength with a smaller liver volume in the whole population, as well as in both men and women. In the whole population, a 1 L/min lower VO_2peak_ was associated with a 0.15 cm^3^ (95% confidence interval [CI]: 0.11 to 0.19; *P* < 0.0001) smaller liver volume for both sexes together. Similarly, a 1 kg lower handgrip strength was associated with a 7.05 cm^3^ (95% CI: 4.87 to 9.23; *P* < 0.001) smaller liver volume in the whole population.

**Conclusion:**

Our results derived from a large community-based sample showed that lower values of VO_2peak_ and handgrip strength were associated with a smaller liver volume. These results might explain the possible negative effects of sedentary lifestyle on liver volume – the sedentary liver.

## Introduction

Physical fitness is considered to be a significant predictor of mortality and morbidity (1), and can be separated in cardiorespiratory fitness and muscular fitness (2). While cardiorespiratory fitness refers to the ability of the cardiovascular and respiratory systems to afford constant oxygen and blood supply to skeletal muscles during exercise (3), muscular fitness indicates the muscular mass and the physical strength, power, and endurance (4). Cardiorespiratory fitness is measured by peak oxygen uptake (VO_2peak_) through cardiopulmonary exercise test (5), while muscular fitness can be accurately measured by handgrip strength (6, 7). To date, VO_2peak_ is considered the gold standard method for the estimation of cardiorespiratory fitness and the better predictor of cardiovascular mortality and morbidity (8). Lower muscular strength is a predictor of ageing and overall poor health outcome (9). The capacity of muscular strength and power decreases with ageing and is associated with physical disability and mortality (10). While VO_2peak_ may predict the risk for cardiovascular events and mortality better than handgrip strength (11), handgrip strength is suggested to be a simple and independent prognostic marker for cardiovascular and all-cause mortality in the population-based on data taken from 17 countries (12).

Sedentary behaviour is increasing in the general population of Western countries, and is an established risk factor for cardiovascular disease and all-cause mortality (13). Physical inactivity is also a risk factor for all types of advanced liver diseases and particularly for hepatic steatosis (14). A recent meta-analysis showed that an increase in VO_2peak_ and a decrease in body weight are both associated with a decrease in liver fat content as assessed by either magnetic resonance imaging (MRI) or proton magnetic resonance spectroscopy (15). Similarly, lower handgrip strength is associated with higher serum liver enzyme levels and higher risk of liver diseases in adolescents, especially in boys (16). Recent cross-sectional studies published by our group (17–20) showed that low values of cardiorespiratory (17, 18, 20) and muscular fitness (19) were significantly associated with a smaller and stiffer heart, which we called ‘the sedentary heart’. Previous studies investigated associations of cardiorespiratory fitness (21) or handgrip strength (22) with hepatic steatosis, but there is currently a lack of published studies investigating the associations of cardiorespiratory fitness or handgrip strength with MRI-assessed liver volume. A recent study (23) from our group showed a nearly three-fold higher risk of all-cause mortality in individuals with a larger liver volume independently of known metabolic risk factors. In a similar way, we aimed to investigate whether markers of cardiorespiratory fitness and muscular fitness are associated with liver volume. Therefore, we examined the associations of cardiorespiratory fitness and muscular fitness determined by VO_2peak_ and handgrip strength with MRI-assessed liver volume in a population-based cohort from Germany.

## Materials and methods

### Study population

**Figure 1:**
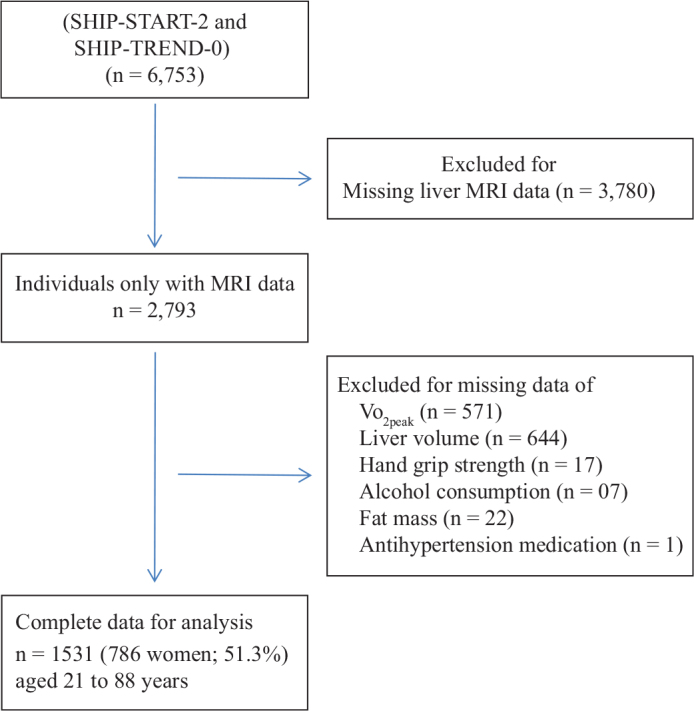
Flow chart for the study participants.

The data were obtained from the Study of Health in Pomerania (SHIP), a population-based study conducted in Northeast Germany. The study design and recruitment strategy have been described elsewhere in more detail (24). For the current analysis, we used data from participants of the SHIP-START-2 and SHIP-TREND-0 (2008–2012). From the 6,753 adult individuals participating in the SHIP-START-2 or SHIP-TREND-0, we used a subsample of 2,793 subjects who were eligible and underwent a whole-body MRI. From this sample we excluded individuals with missing values in VO_2peak_ (*n* = 571), liver volume (*n* = 644), handgrip strength (*n* = 17), body fat mass (*n* = 22), alcohol consumption (*n* = 7), and use of antihypertensive medication (*n* = 1). Thus, the final sample of participants consisted of 1,531 subjects (786 women; 51.3%) aged 21 to 88 years (Figure 1).

All participants have given written informed consent to participate in the study. The study conformed to the ethical guidelines of the Declaration of Helsinki as reflected in *a priori* approval by the local ethics committee of the University of Greifswald.

### Cardiopulmonary exercise testing and gas exchange variables

A symptom-limited exercise test using a calibrated electromagnetically braked cycle ergometer (Ergoselect 100, Ergoline, Germany) was performed in all participants according to a modified Jones protocol. Oxygen uptake (VO_2_) was analysed breath-by-breath averaged over 10-sec intervals using the Oxycon Pro system (Jaeger/Viasys Healthcare; Hoechberg, Germany), together with a Rudolph’s mask, which was recalibrated before each test. VO_2peak_ in L/min was defined as the highest 10-sec average of absolute VO_2_ during late exercise or early recovery (25).

### Handgrip strength assessment

Handgrip strength was measured one time in each hand for all the participants using a hand-grip dynamometer (Smedley’s Dynamometer, Scandidact, Odder, Denmark), with the upper arm being held against the trunk and the elbow in a 90-degree flexion while standing. Participants were instructed to squeeze the handle as hard as possible for 3 sec, and the maximum contractile force in kilogram (kg) was recorded. The highest value obtained from the left and right handgrip strength measurements was used in the present analyses (19).

### Bioelectrical impedance analysis

Fat-free mass and fat mass were measured by bioelectrical impedance analysis using a multifrequency Nutriguard-M device (Data Input, Pöcking, Germany) and the NUTRI4 software (Data Input, Pöcking, Germany) (26).

### Magnetic resonance imaging

A liver MRI was performed in all the participants using a 1.5-Tesla MRI system (Magnetom Avanto, software version VB15; Siemens Healthineers Erlangen, Germany) with a 12-channel phased-array surface coil 25. Liver fat content was assessed with offline reconstructions of a proton density fat fraction (PDFF) map. Mean PDFF values were determined at operator-defined regions of interest placed at the centre of the liver, by using Osirix (v3.8.1; Pixmec Sarl, Bernex, Switzerland). Presence of hepatic steatosis was defined as MRI-PDFF ≥5.1% (27).

### Assessment of liver volume

Assessment of liver volume was performed by a calculation of volume indices. For this reason, measurement of liver diameters was performed using the software Osirix (version 4.6; Pixameo, Bernex, Switzerland) by a trained observer who was blind to clinical and biochemical data of participants. In each participant, MRI-assessed liver diameters were measured in mid-clavicular line in the following three orientations: anterior-posterior (1); lateral-medial (2); and cranio-caudal (6). Thereafter, the volume index and estimated liver volume was calculated using the following formula*: liver volume = (A × B × C)/2.6* (28)*.*

### Statistical analysis

**Figure 2:**
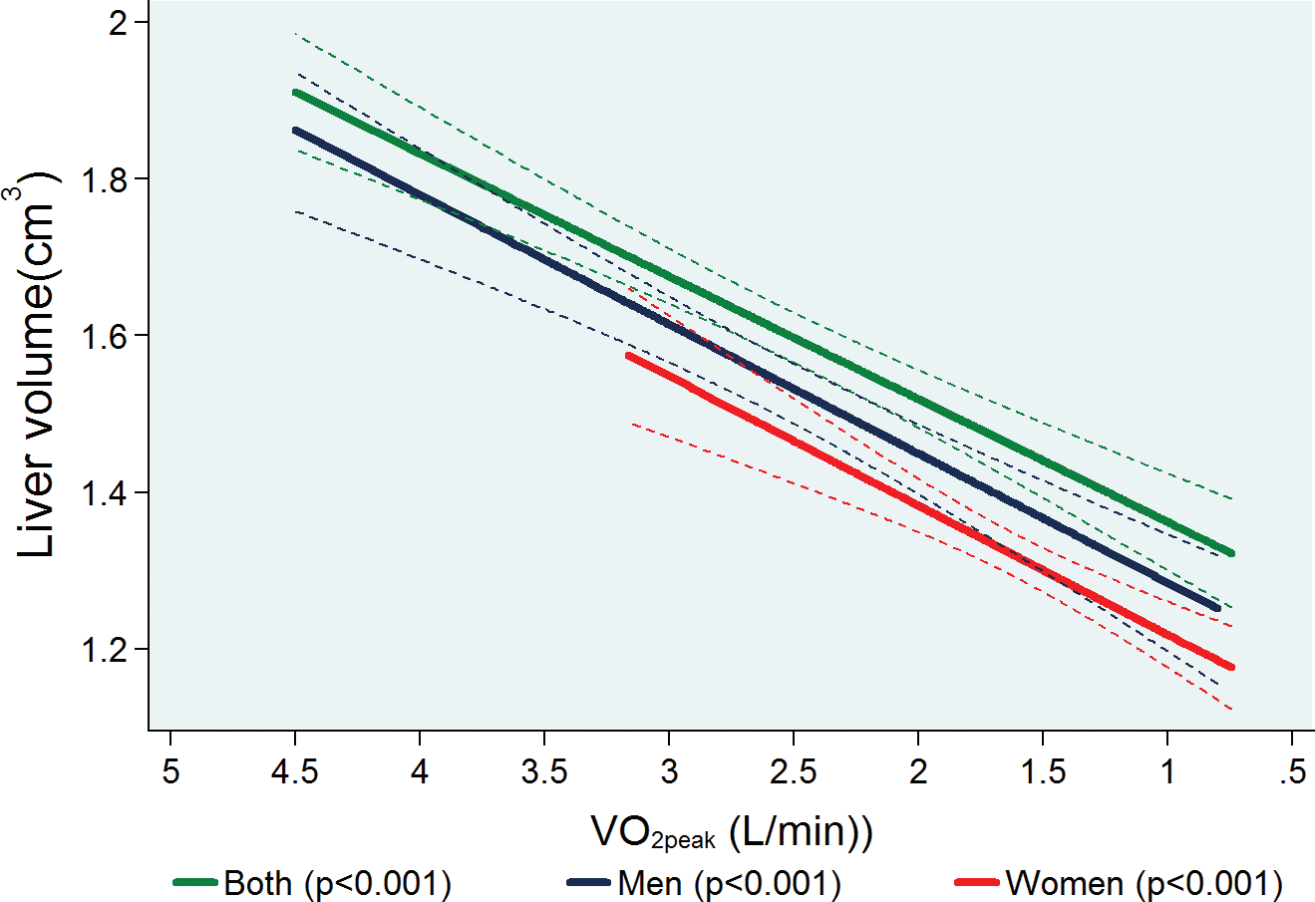
Adjusted* regression line showing the associations between peak oxygen uptake (VO_2peak_) and MRI-assessed liver volume in both pooled sex analyses (*n* = 1,531) and stratified by sex (men = 745; women = 786). *Linear regression was adjusted for age, sex (except when stratified by sex), body fat mass, pre-existing type 2 diabetes, daily alcohol consumption, and smoking status use of hypoglycaemic or antihypertensive medications. **NB:** The association between VO_2peak_ and liver volume is presented with a reverse x-axis to make the interpretation of results more intuitive.

Continuous data were expressed as medians (with 25th percentiles and 75th percentiles) and categorical variables as absolute and relative percentages by quartiles of VO_2peak_ and sex. Multivariable linear regression models were used for testing the associations of VO_2peak_ and handgrip strength with liver volume. These regression models were adjusted for age, sex (except when stratified by sex), body fat mass, pre-existing type 2 diabetes, daily alcohol consumption, smoking status, and use of hypoglycaemic or antihypertensive medications (Figure 2).

A *P* < 0.05 was considered to be statistically significant. All statistical analyses were performed using Stata 14.2 (Stata Corporation, College Station, TX, USA).

## Results

Table 1 and Table 2 shows descriptive data of the study participants stratified by VO_2peak_ quartiles and sex. In both men and women, the median VO_2peak_, handgrip strength and total body weight increased from the first to the fourth quartile of VO_2peak_. Men and women in the first VO_2peak_ quartile were older_,_ had higher systolic blood pressure, and were more likely to have hypertension and known diabetes with a more frequent use of hypertensive, glucose-lowering, and lipid-lowering medications than subjects belonging to other VO_2peak_ quartiles. Men in the VO_2peak_ fourth quartile were more likely to be (former and current) smoker compared to men in the other quartiles.

**Table 1:** Characteristics of study sample stratified by VO_2peak_ quartiles in men (*n* = 745).

Parameter(s)	First quartile (*n* = 187)	Second quartile (*n* = 186)	Third quartile (*n* = 186)	Fourth quartile (*n* = 186)	*P*
VO_2peak_ (L/min)	1.76 (1.56; 1.90)	2.20 (2.10; 2.35)	2.63 (2.53; 2.75)	3.16 (3.00; 3.41)	< 0.001
Handgrip strength (kg)	41.0 (36.0; 47.0)	47.0 (41.5; 52.0)	49.5 (44.5; 54.0)	52.0 (47.0; 57.5)	< 0.001
Liver volume (cm^3^)	1.41 (1.25; 1.63)	1.55(1.35; 1.74)	1.65(1.40; 1.91)	1.69 (1.50; 1.91)	< 0.001
Age (years)	65.5 (56.0; 72.0)	55.0 (46.0; 64.0)	49.5 (40.0; 57.0)	42.0 (36.0; 49.0)	< 0.001
Height (cm)	173 (170; 178)	175 (171; 180)	178 (174; 181)	181 (177; 185)	< 0.001
Waist circumference (cm)	97.0 (90.0; 104)	95.5 (89.5; 104)	94.5 (87.5; 103)	91.0 (84.0; 100)	< 0.001
Waist-to-height ratio	0.56 (0.52;60.0)	0.55 (0.51; 0.59)	0.53 (0.50; 0.58)	0.50 (0.46; 0.56)	< 0.001
Body mass index (kg/m^2^)	27.5 (25.7; 30.1)	27.8 (25.9; 30.5)	27.8 (25.5; 30.5)	27.0 (24.8; 30.2)	0.154
Body fat-free mass (kg)	63.3 (58.2; 68.4)	65.4(61.0; 71.0)	67.0 (62.0; 72.3)	69.2 (64.1; 74.6)	< 0.001
Body fat mass (kg)	20.0 (16.0; 24.6)	20.2 (16.5; 25.1)	20.4 (16.0; 25.0)	19.4 (15.1; 25.5)	0.796
Total body weight (kg)	82.0 (75.3; 92.4)	86.2 (78.1; 96.0)	87.1 (80.0; 96.6)	88.7 (80.2; 99.2)	< 0.001
Systolic blood pressure (mmHg)	135 (125; 146)	134 (124; 146)	134 (124; 144)	128 (121; 138)	< 0.001
Diastolic blood pressure (mmHg)	79 (72; 86)	81 (75; 88)	82 (76; 89)	78 (73; 84)	< 0.001
Hypertension (%)	64.5	57.4	52.3	31.5	< 0.001
Antihypertensive medications (%)	42.6	32.3	26.1	13.2	<0.001
Glycated haemoglobin (%)	5.40 (5.20; 5.80)	5.30 (5.00; 5.70)	5.30 (5.10; 5.60)	5.20 (4.90; 5.40)	< 0.001
Pre-existing type 2 diabetes (%)	17.6	10.7	4.30	1.08	< 0.001
Hypoglycaemic medications (%)	12.8	8.60	3.23	0.54	< 0.001
LDL-cholesterol (mmol/L)	3.36 (2.84; 3.87)	3.46 (2.88; 4.01)	3.48 (3.01; 4.04)	3.33 (2.67; 3.89)	0.008
HDL-cholesterol (mmol/L)	1.24 (1.05; 1.45)	1.26 (1.08; 1.49)	1.28 (1.07; 1.48)	1.28 (1.09; 1.55)	0.216
Lipid lowering medications (%)	21.6	16.9	8.09	2.63	< 0.001
Smoking (%)					
Never	27.3	28.1	32.6	39.7	0.067
Former	46.7	46.2	45.5	41.3	
Current	26.0	25.6	22.0	19.0	
Alcohol consumption (g/day)	6.82 (1.88; 16.9)	9.22 (3.43; 19.7)	9.83 (3.74; 19.7)	9.06 (3.07; 19.3)	0.058
Sedentary lifestyle (%)	36.5	31.0	31.4	19.0	< 0.001

**Table 2:** Characteristics of study sample stratified by VO_2peak_ quartiles in women (*n* = 786).

Parameter(s)	First quartile (*n* = 197)	Second quartile (*n* = 196)	Third quartile (*n* = 197)	Fourth quartile (*n* = 196)	*P*
VO_2peak_ (L/min)	1.17 (1.05; 1.27)	1.46 (1.40; 1.50)	1.68 (1.62; 1.75)	2.00 (1.90; 2.20)	< 0.001
Handgrip strength (kg)	25.0 (21.5; 28.5)	27.0 (23.5; 31.0)	28.0 (25.0; 31.0)	31.0 (28.0; 35.0)	< 0.001
Liver volume (cm^3^)	1.20 (1.05; 1.36)	1.24 (1.22; 1.43)	1.31 (1.18; 1.48)	1.45 (1.30; 1.64)	< 0.001
Age (years)	62.0 (52.0; 70.0)	56.0 (46.0; 62.0)	49.0 (42.0; 59.0)	44.0 (39.0; 52.0)	< 0.001
Height (cm)	160 (157; 165)	163 (158; 167)	164 (160; 169)	167 (164; 171)	< 0.001
Waist circumference (cm)	82.3 (75.0; 91.5)	81.4 (74.4; 91.8)	81.5 (74.7; 92.4)	82.0 (73.9; 92.4)	0.878
Waist-to-height ratio	0.51 (0.46; 0.57)	0.50 (0.46; 0.57)	0.50 (0.45; 0.56)	0.49 (0.49; 0.56)	0.074
Body mass index (kg/m^2^)	26.0 (23.0; 29.3)	26.1 (23.5; 30.0)	25.9 (23.3; 30.2)	26.9 (24.0; 31.0)	0.015
Body fat-free mass (kg)	45.0 (41.8; 47.6)	45.5 (42.7; 49.0)	47.4 (44.3; 50.5)	50.3 (47.1; 54.6)	< 0.001
Body fat mass (kg)	23.0 (16.5; 28.5)	23.4 (18.0; 30.0)	23.4 (18.2; 29.7)	26.0 (19.5; 33.1)	< 0.001
Total body weight (kg)	66.7 (60.0; 75.2)	68.5 (61.5; 78.1)	71.0 (64.0; 80.3)	76.0 (66.7; 86.6)	< 0.001
Systolic blood pressure (mmHg)	125 (112; 137)	120 (110; 132)	117 (109; 129)	116 (108; 127)	< 0.001
Diastolic blood pressure (mmHg)	73 (68; 81)	75 (69; 81)	75 (69; 81)	74 (69; 81)	0.442
Hypertension (%)	48.1	38.5	30.9	26.7	< 0.001
Antihypertensive medications (%)	35.6	28.4	24.2	21.7	< 0.002
Glycated haemoglobin (%)	5.30 (4.90; 5.60)	5.20 (4.90; 5.60)	5.20 (4.80; 5.50)	5.10 (4.80; 5.50)	0.004
Pre-existing type 2 diabetes (%)	9.64	8.67	5.58	3.57	0.026
Hypoglycaemic medications (%)	7.61	4.08	2.54	0.51	0.002
LDL-cholesterol (mmol/L)	3.48 (2.92; 4.08)	3.38 (2.78; 4.11)	3.23 (2.68; 3.87)	3.14 (2.48; 3.79)	< 0.001
HDL-cholesterol (mmol/L)	1.61 (1.36; 1.91)	1.61 (1.36; 1.92)	1.60 (1.38; 1.80)	1,57 (1.32; 1.83)	0.238
Lipid lowering medications (%)	15.9	10.7	4.60	2.04	< 0.001
Smoking (%)					
Never	56.2	47.5	43.1	41.0	0.010
Former	24.8	30.7	36.5	38.2	
Current	19.0	21.8	20.4	20.9	
	1.80 (0.17; 5.02)	2.56 (0.72; 5.22)	2.74 (0.72; 6.61)	2.90 (0.99; 6.52)	< 0.001
	31.4	28.8	24.2	18.1	0.003

Data are presented as medians (25th, 75th percentiles) for continuous data and absolute numbers or percentages for categorical data. Kruskal-Wallis test was used for continuous variables and chi- square tests for the categorical variables.

### Reversal of the x-axis scale to run from maximum VO2peak and handgrip strength value to minimum

Sedentary lifestyle is significantly associated with lower values of both VO_2peak_ and handgrip strength. The central effort of our study is to show the results of consecutively lower values of both VO_2peak_ and handgrip strength on liver volume after adjusting for potential confounding factors. For such reason, all associations between VO_2peak_, and handgrip strength with liver volume are presented with a reverse x-axis to make the interpretation of results more intuitive.

### Association between VO2peak and liver volume

Figure 2 shows the adjusted linear association between VO_2peak_ and liver volume after adjustment for potential confounding factors. In multivariable adjusted regression analyses, we observed a significant association between lower VO_2peak_ and smaller liver volume for both sexes together and also separately for men and women when stratified by sex. In detail, a 1 L/min lower VO_2peak_ was associated with a 0.15 cm^3^ (95% confidence interval [CI]: 0.12 to 0.19; *P* < 0.001) lower liver volume for both sexes together. When stratified by sex, a 1 L/min lower VO_2peak_ was associated with a 0.16 cm^3^ (0.11 to 0.21; *P* < 0.001) lower liver volume in men and a 0.17 cm^3^ (0.12 to 0.23; *P* < 0.001) in women, respectively (Table 3).

**Table 3:** Sex-stratified associations of handgrip strength and VO_2peak_ with liver volume.

Variables	Men			Women		
	ß (95% CI)	*P*	Adjusted R^2^	ß (95% CI)	*P*	Adjusted R^2^
Handgrip strength (kg)	7.73 (4.67; 10.8)	0.000	0.38	4.52 (0.97; 8.06)	0.01	0.42
VO_2peak_ (L/min)	0.16 (0.11; 0.20)	0.000	0.40	0.17 (0.11; 0.23)	0.000	0.45

CI: Confidence intervals.

Linear regression was used by calculating beta coefficient with 95% CI and was adjusted for age, body fat mass, pre-existing type 2 diabetes, daily alcohol consumption and use of hypoglycaemic or antihypertensive medications.

ß (95% CI): beta-coefficient with 95% confidence intervals; VO_2peak_: peak oxygen uptake.

### Association between handgrip strength and liver volume

**Figure 3:**
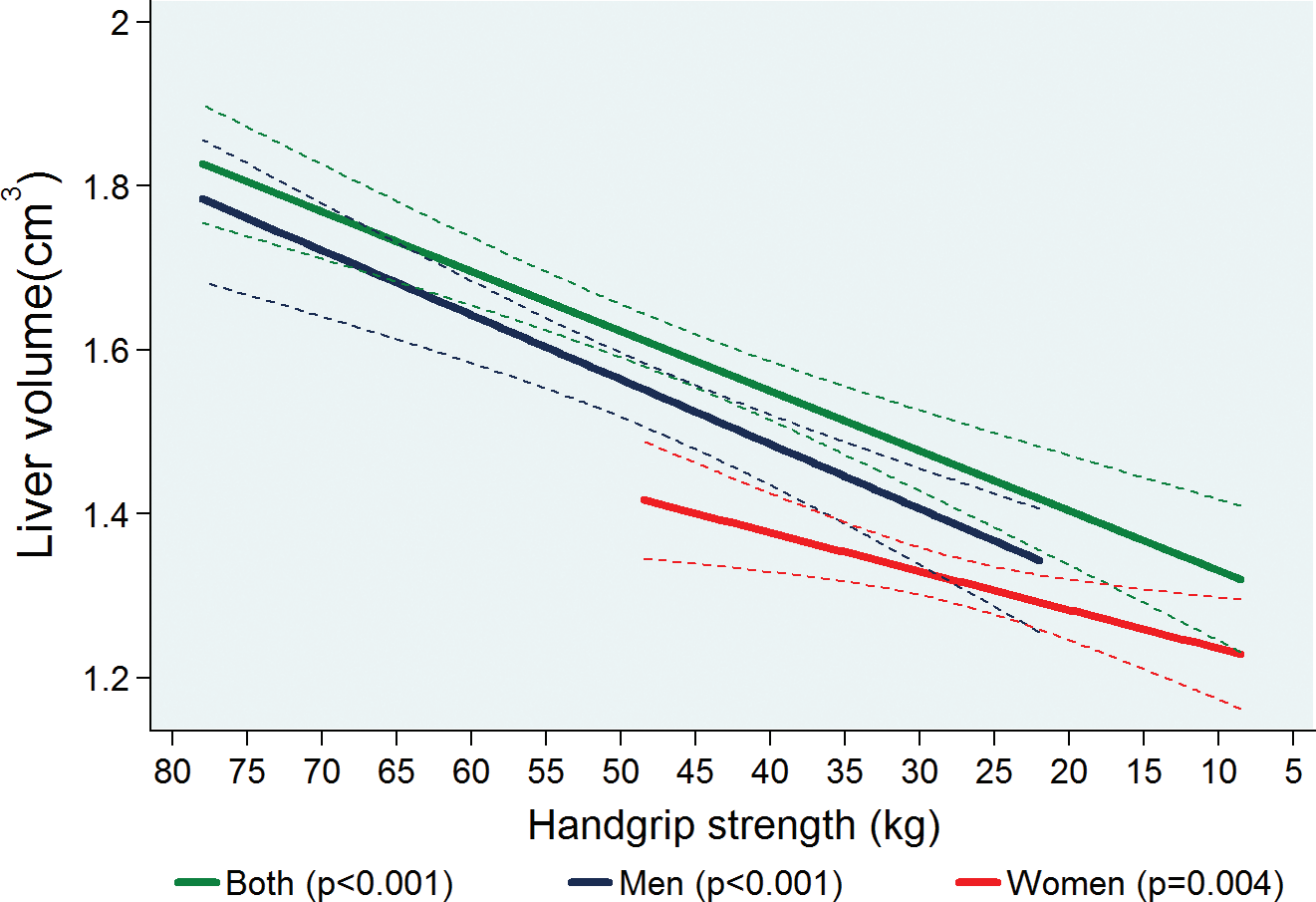
Adjusted* regression line showing the associations between handgrip strength and MRI-assessed liver volume in both pooled sex analyses (*n* = 1,531) and stratified by sex (men = 745; women = 786). *Linear regression was adjusted for age, sex (except when stratified by sex), body fat mass, pre-existing type 2 diabetes, daily alcohol consumption, and use of hypoglycaemic or antihypertensive medications. **NB:** The association between handgrip strength and liver volume is presented with a reverse x-axis to make the interpretation of results more intuitive.

There was a significant adjusted association between lower handgrip strength and smaller liver volume for both sexes and also separately for men and women when stratified by sex (Figure 3). A 1-kg lower handgrip strength was associated with a 7.28 cm^3^ (95% CI: 5.16 to 9.42; *P* < 0.001) lower liver volume for both sexes together. When stratified by sex, a 1 kg lower handgrip strength was associated with a 7.73 cm^3^ (4.67 to 10.8; *P* < 0.001) lower liver volume in men and a 4.52 cm^3^ (0.97 to 8.06; *P* = 0.01) in women, respectively (Table 3).

We standardised VO_2peak_ and handgrip strength and investigated their associations with liver volume. A one standard deviation (SD) lower VO_2peak_ was associated with a 0.32 cm^3^ (95% CI: 0.25 to 0.38) smaller liver volume while a one SD lower handgrip strength was associated with a 0.09 cm^3^ (95% CI: 0.06 to 0.11) smaller liver volume.

We further investigated the potential effect modifications of study cohort (SHIP-START-2 vs. SHIP-TREND-0) on the observed associations between VO_2peak_ or handgrip strength with liver volume. In none of these subgroup analyses, we observed any significant interaction between the study cohort and the respective exposure on liver volume (*P* > 0.05). In sensitive analysis, we found that the fat-free mass (FFM) explains 55% variation in liver volume and 47% in VO_2peak_ and handgrip strength.

## Discussion

As far as we know, this is the first and largest population-based study investigating the associations of VO_2peak_ and handgrip strength with MRI-assessed liver volume. In our cohort of 1,531 German middle-aged individuals, we found that lower values of VO_2peak_ and handgrip strength were significantly associated with a smaller liver volume in the whole population as well as in men and women, separately. These associations remained statistically significant even after adjustment for potential confounding factors, such as age, sex (except when stratified by sex), body fat mass, pre-existing type 2 diabetes, daily alcohol consumption, smoking history, and current use of hypoglycaemic or antihypertensive medications.

The putative biological mechanisms underpinning our observed associations are not entirely clear, but a possible explanation might be a decrease in FFM following a sedentary lifestyle. A decrease in FFM might lead to a reduction in total blood and plasma volume. While an increase in physical activity or exercise training raises the total blood and plasma volume (29), results from bed deconditioned athletes, and bed rest experiments showed that decrease in physical activity can lead to a reduction in total blood and plasma volume (30). A previous study showed that FFM may account for nearly 80 to 85% of the variance in the directly measured whole blood or plasma volumes, respectively (31). We performed sensitivity analyses showing that most of the variation in liver volume was explained by FFM, which constitutes approximately 50% of skeletal muscle mass (32). Fat free mass is considered the main factor for changes in blood and plasma volumes through its metabolic demands (33). The reduction in blood and plasma volumes will result in a lower volume unload of the liver with a possible consequent dwindling in its size. Moreover, a more sedentary lifestyle may also lead to a slower and gradual reduction in total skeletal muscle mass, further reducing physical activity (34) (Figure 3).

A previous study from our group found that lower cardiorespiratory fitness measured by Vo_2peak_ was associated with higher liver fat content and gamma-glutamyl transferase (GGT) levels, particularly in obese and overweight individuals (21). This finding may be explained by effects of sedentary lifestyle on body fat, which may result in a larger liver fat content, but not necessarily in a larger liver volume. Thus, the results of the present study are not in contrast to our previous study as effects of VO_2peak_ on relative fat content and liver function may differ to those on absolute liver volume. Contrary to this findings, we previously demonstrated that a larger liver volume is associated with a 3-fold higher risk of all-cause mortality. The possible explanation for these different results might be that larger liver volume is often associated with hepatic steatosis and fibrosis, which contribute to metabolic dysfunction and increased mortality risk (35). In contrast, a previous study showed that decreased skeleton muscle mass and strength are associated with poor hepatic function and metabolic health (36). Furthermore, poor physical fitness is associated with reduced blood flow to the liver and muscle-liver crosstalk dysfunction, which might contribute to a smaller liver volume (37).

After standardisation of the VO_2peak_ and handgrip strength values, the VO_2peak_ showed a stronger association with liver volume than handgrip strength . However, it is more difficult to improve the VO_2peak_ by endurance training than to increase handgrip strength by resistance training. Thus, for lifestyle intervention we believe that it may be favourable to increase handgrip strength to prevent a decline in liver volume.

One of the major strengths of our study is that it includes a large number of individuals derived from a population-based study. In addition, liver volume was assessed by using MRI-PDFF, which is the most accurate and sensitive marker to measure liver volume compared to other non-invasive methods such as ultrasound (38). Along with this, we measured VO_2peak_ by a standard method of the cardiopulmonary exercise test.

Limitations of our study include its cross-sectional design. Hence, we cannot draw any causal inference from the observed associations. Second, we included only Caucasian individuals in our analysis; therefore, the generalisability of our results to other ethnic groups might be not appropriate. Finally, although we adjusted our regression models for potential confounding factors, there is still the possibility of unmeasured or unknown potential confounders.

## Conclusion

Our results derived from a large community-based sample showed that lower values of VO_2peak_ and handgrip strength are closely associated with a smaller liver volume (as assessed by MRI). These results might explain the possible adverse effects of sedentary lifestyle on liver volume – the sedentary liver.

## ORCID

Muhammad Naeem https://orcid.org/0000-0001-8455-6599

Beate Stubbe https://orcid.org/0000-0002-7571-744X

Ralf Ewert https://orcid.org/0000-0002-6746-9925

Robin Bülow https://orcid.org/0000-0003-1884-5784

Martin Bahls https://orcid.org/0000-0002-2016-5852

Marcus Doer https://orcid.org/0000-0001-7471-475X

Ali Aghdassi https://orcid.org/0000-0002-0569-7316

Giovanni Targher https://orcid.org/0000-0002-4325-3900

Henry Völzke https://orcid.org/0000-0001-7003-399X

Marcello RP Markus https://orcid.org/0000-0002-6234-4955

Till Ittermann https://orcid.org/0000-0002-0154-7353
